# PP27 Health Technology Assessment For Obesity Treatment In The Brazilian Public Health System: A Review Of Technology Incorporation Decisions

**DOI:** 10.1017/S0266462325102468

**Published:** 2025-12-29

**Authors:** Jaqueline Horvath, Barbara Medeiros Silva, Maria Angélica Pires Ferreira

## Abstract

**Introduction:**

Obesity is increasing at an alarming rate in Brazil. Beyond its strong association with chronic diseases, obesity imposes a substantial burden on the Brazilian Unified Health System (SUS). The incorporation of new treatments, including interventions for weight management, requires rigorous assessment of efficacy, safety, and cost effectiveness by the National Committee for Health Technology Incorporation (CONITEC).

**Methods:**

This study aimed to review decisions regarding the incorporation of health technologies for obesity treatment into the SUS. A review of CONITEC recommendations was performed, utilizing reports publicly accessible on its official website. The analysis focused on the evaluation of pharmacological and procedural technologies for obesity treatment in Brazil, examining technical and economic impact assessment reports.

**Results:**

Between 2012 and 2022, four technologies were evaluated for obesity treatment: sibutramine, orlistat, bariatric surgery via video laparoscopy (all requested by the Ministry of Health and medical societies), and liraglutide 3 mg (requested by a pharmaceutical company). Among these, only bariatric surgery was incorporated into the SUS in 2017. Sibutramine and orlistat were rejected due to limited efficacy and unfavorable safety profiles. The evaluation of liraglutide, aimed at patients with a body mass index above 35 kg/m² and cardiovascular risk, demonstrated efficacy, but was declined due to its high cost and significant economic impact on the SUS.

**Conclusions:**

The rejection of technologies by the SUS highlighted safety, efficacy, and cost concerns. These decisions aligned with the SUS priority to remain economically sustainable while promoting multidisciplinary approaches to obesity management, which are actively offered within the SUS. Given the importance of the condition for public health, it is considered that the topic has been under-prioritized in the incorporation process within the SUS.

